# Genome sequence of *Mesorhizobium mediterraneum* strain R31, a nitrogen-fixing rhizobium used as an inoculant for chickpea in Argentina

**DOI:** 10.1128/MRA.00581-23

**Published:** 2023-09-29

**Authors:** Emiliano Foresto, Santiago Revale, Fiorela Nievas, María Evangelina Carezzano, Mariana Puente, Pedro Alzari, Mariano Martinez, Mathilde Ben-Assaya, Damien Mornico, Maricel Santoro, Francisco Martinez-Abarca, Walter Giordano, Pablo Bogino

**Affiliations:** 1 Departamento de Biología Molecular, Instituto de Biotecnología Ambiental y Salud (INBIAS-CONICET), Facultad de Ciencias Exactas, Físico-Químicas y Naturales, Universidad Nacional de Río Cuarto, Río Cuarto, Córdoba, Argentina; 2 Wellcome Centre for Human Genetics, University of Oxford, Oxford, United Kingdom; 3 Instituto de Microbiología y Zoología Agrícola, Instituto Nacional de Tecnología Agropecuaria (IMYZA-INTA), Castelar, Argentina; 4 Unité de Microbiologie Structurale, Institut Pasteur, CNRS UMR 3528, Université de Paris, Paris, France; 5 Département Biologie Computationnelle, Hub de Bioinformatique et Biostatistique, CNRS USR 3756, Institut Pasteur, Paris, France; 6 Department of Biochemistry, Max Planck for Chemical Ecology, Jena, Germany; 7 Grupo de Ecología Genética de la Rizósfera, Estación Experimental del Zaidín (CSIC), Granada, Spain

**Keywords:** *Mesorhizobium mediterraneum*

## Abstract

Here, we report the complete genome sequence of *Mesorhizobium mediterraneum* R31, a rhizobial strain recommended and used as a commercial inoculant for chickpea in Argentina. The genome consists of 7.25 Mb, distributed into four circular replicons: a chromosome of 6.72 Mbp and three plasmids of 0.29, 0.17, and 0.07 Mbp.

## ANNOUNCEMENT

Inoculating legumes with rhizobia is a sustainable agricultural strategy. Rhizobia fertilize the crop biologically through nitrogen-fixing symbiosis and thus prevent nitrogen deficiency (1, 2). The Instituto Nacional de Tecnología Agropecuaria (INTA) in Argentina provides two strains of different species to inoculate chickpea, *Mesorhizobium ciceri* R30 and *Mesorhizobium mediterraneum* R31 (3). We recently reported the complete annotated genome of the former, *M. ciceri* strain R30 (4); here, we announce the complete annotated genome of the latter, *M. mediterraneum* R31. To date, only two incomplete genomes for *M. mediterraneum* strains (CCBAU 01399 and USDA 3392) are available in NCBI.

A pure culture of *M. mediterraneum* R31, grown in yeast extract-mannitol medium at 30°C with 150 rpm rotation for four4 days (optical density at 600 nm [OD_600_], 1.0) (5), was the source for the total DNA. It was obtained with a DNeasy Blood && Tissue Kit (Qiagen) for Illumina sequencing and a Promega Wizard HMW DNA Extraction Kit (Promega) for Oxford Nanopore Technologies sequencing. The genome was assembled through a hybrid approach, including short Illumina reads and long Oxford Nanopore reads. Illumina’s library was prepared with a Nextera XT DNA Library Preparation Kit and sequenced on Illumina NextSeq 500 with a paired-end 150-bp read configuration. Nanopore’s library was prepared with an Oxford Nanopore Technologies Rapid Barcoding sequencing kit (SQK-RBK004) and sequenced in two Flongle flowcells. Data were base-called on Guppy v4.2.2, using the high-accuracy model and the --trim_barcodes option. We obtained 6,441,624 Illumina PE reads predicting a 132-fold coverage, and 119,523 Nanopore long reads which averaged 5,027 bp and predicted an 83-fold coverage. The raw reads were quality controlled by manually inspecting the reports obtained on FastQC (6) (Illumina) and PycoQC (7) (Nanopore). Hybrid genome assembly was performed with the raw reads in the nf-core/bacass pipeline (commit ceebac0) set at default parameters (8). This resulted in four contigs that were closed by manually analyzing the overlapped ends on Geneious software version 2019 2.1 (9). DNA content analysis revealed a genome made up of one large chromosome (6,720,795 bp, G+C content 62.2%) and three minor replicons from the repABC plasmid family (10) (293,882 bp, G+C content 60.1%; 175,478 bp, G+C content 60.2%; and 66,723 bp, G+C 58.6%).

The complete genome, which was annotated in the NCBI Prokaryotic Genomes Annotation Pipeline (PGAP) (11–13), consists of 6,695 protein-coding sequences, two complete ribosomal operons, and 53 tRNAs. The genes for nodulation (*nod*) and nitrogen fixation (*nif* and *fix*) appear to be located on a chromosomal 480 kb symbiosis island (1,850,730 to 2,330,771 bp), flanked by direct repeat sequences (22 nt) identical to those in the ICE region in other mesorhizobia, and adjacent to one of four serine tRNA genes (4, 14). This region also harbors mobile genes (transposases, integrases, and recombinases) probably associated with the island’s excision and transfer (15, 16).

This complete genome sequence of an *M. mediterraneum* strain could be crucial for more in-depth research into its symbiotic performance and other biological features of this species.

## Data Availability

The complete genome sequence of *Mesorhizobium mediterraneum* R31 is available at NCBI GenBank under accession numbers CP088151 for the chromosome, CP088152, CP088153, and CP088154 for the plasmids with BioProject ID PRJNA782313. and BioSample accession SAMN23372083. Raw data reads are available at NCBI’s Sequence Read Archive under accession numbers SRR16993324 to SRR16993326.
